# Celecoxib inhibits acute edema and inflammatory biomarkers through peroxisome proliferator-activated receptor-γ in rats

**DOI:** 10.22038/ijbms.2020.43995.10315

**Published:** 2020-12

**Authors:** Gholamreza Houshmand, Bahareh Naghizadeh, Behnam Ghorbanzadeh, Zahra Ghafouri, Mehdi Goudarzi, Mohammad Taghi Mansouri

**Affiliations:** 1 Department of Pharmacology, School of Medicine, Mazandaran University of Medical Sciences (MAZUMS), Sari, Iran; 2 Department of Anesthesiology, College of Physicians and Surgeons, Columbia University, New York, NY 10032, USA; 3 Department of Pharmacology, School of Medicine, Dezful University of Medical Sciences, Dezful, Iran; 4 Department of Biochemistry Biophysics and Genetics, School of Medicine, Mazandaran University of Medical Sciences (MAZUMS), Sari, Iran; 5 Medicinal Plant Research Center, Ahvaz Jundishapur University of Medical Sciences, Ahvaz, Iran; 6 Toxicology Research Center, Ahvaz Jundishapur University of Medical Sciences, Ahvaz, Iran

**Keywords:** Carrageenan, Celecoxib, Cytokines, Oxidative stress, Pioglitazone, PPAR-γ, Rat

## Abstract

**Objective(s):**

Celecoxib (CLX), a selective cyclooxygenase-II (COX-2) inhibitor, has been used for management of several inflammatory disorders. The present study aimed to explore the role of peroxisome proliferator-activated receptor-gamma (PPARγ) in CLX induced anti-inflammatory response in rats.

**Materials and Methods:**

Carrageenan-induced paw edema was used as an acute inflammation model. Rats were treated with various intra-peritoneal (IP) doses of CLX (0.3–30 mg/kg) and pioglitazone (PGL; PPARγ agonist, 1–20 mg/kg) alone or in combination. Amounts of PPARγ, COX-2, and prostaglandin E2 (PGE2) in paw tissue, and extents of TNF-α and IL-10 in serum were measured. Moreover, levels of oxidative stress parameters as malondialdehyde (MDA), glutathione (GSH), glutathione peroxidase (GPx) activity in the cortex, hippocampus, and paw tissues were also determined.

**Results:**

CLX and PGL dose-dependent administration (IP), alone or in combination reduced carrageenan-induced paw edema. Further, both agents, alone or in combination, reduced either the amounts of COX-2, PGE2, and MDA in the inflamed paw, and the levels of TNF-α in serum which were elevated by carrageenan. Both drugs also increased both levels of PPARγ, GSH, GPx activity in paws, and serum levels of IL-10 that were decreased by carrageenan. Intraplantar injection of GW-9662 (IPL), a selective PPARγ antagonist, inhibited all biochemical modifications caused by both single and combined drug treatments.

**Conclusion:**

CLX produced its anti-inflammatory effects probably through PPARγ receptor activation. Besides, increased anti-inflammatory effects of CLX with PGL suggest that their combination might be applied for the clinical management of inflammation especially in patients suffering from diabetes.

## Introduction

Pathogenesis of various disorders including cancer, aging (1), neurological and cardiovascular diseases (2, 3), diabetes (4), etc., is contingent on inflammation. Moreover, acute inflammation causes the overproduction of free radicals and release of several inflammatory and pro-inflammatory mediators. 

Subplantar administration of carrageenan in rodents produces bi-phasic edema. The first phase, seen in the 1st hr, is connected with the production of histamine, serotonin, bradykinin, and different prostaglandins which are created by cyclooxygenase enzymes (COX), whereas the secondary phase (after 1 hr) is attributed to neutrophil infiltration and constant output of the prostaglandins (5). Release of free radicals and pro-inflammatory cytokines, tumor necrosis factor α (TNF-α) and interleukin-1β (IL-1β) by neutrophils are also implicated in the secondary phase of carrageenan-induced acute inflammation (6). Moreover, it has been determined that oxygen-derived free radicals activate NF-κB (nuclear factor kappa-light-chain-enhancer of activated B cells) which has a role in the synthesis of pro-inflammatory cytokines (7).

Non-steroidal anti-inflammatory drugs (NSAIDs) are broadly administered in the management of inflammatory diseases (8). Although the main hypothesis for NSAIDs mechanism of action is inhibiting COX enzymes, interaction with peroxisome proliferator-activated receptors (PPAR) has been recently proposed (9). Celecoxib (CLX) is the most common NSAID drug that specifically inhibits cyclooxygenase II enzyme (COX-II) (10). There is also much evidence indicating the association between PPARγ receptor activation and COX-2 inhibition/induction in various cell types (11). 

PPARγ, a member of the nuclear receptor superfamily, is recognized as a transcription factor. Its crucial role lies in various physiological functions like lipid and glucose metabolism, and several disorders such as diabetes, dyslipidemia, and hypertension (12, 13). Thiazolidinedione drugs (TZDs) such as pioglitazone (PGL) are a group of anti-diabetics that agonize PPARγ, leading to improvement in glucose metabolism and insulin resistance (14). In addition to metabolic effects, PPAR ligands represent a promising therapeutic strategy associated with inflammatory diseases (15). In our previous study, we reported the anti-inflammatory role of PGL exclusively and cooperatively with indomethacin (IND) in an experimental animal model (16).

The objective of this study was to determine the fundamental mechanisms implicated in association of PPARγ receptors with anti-inflammatory responses produced by CLX using a rat model of carrageenan-induced acute inflammation. 

## Materials and Methods

***Animals***

Six groups of randomly selected adult male Wistar rats (n=7 per group, 230–280 g) with free access to food and water were encased at controlled temperature (22 ± 2 °C). All animal examinations were done in accordance with the National Institutes of Health Guide for Care and Use of Laboratory Animals. The Institutional Animal Ethical Committee of Ahvaz Jundishapur University of Medical Sciences formed under Committee for Purpose of Control and Supervision of Experiments on Animals (CPCSEA, Reg. No. PRC150) approved all protocols. All experiments were done by blinded examiners. Once all tests were done, the animals were euthanized.

***Drugs and chemicals***

Gamma-carrageenan, phenylmethylsulfonyl fluoride (PMSF), aprotinin, and leupeptin were acquired from Sigma-Aldrich Co. (St. Louis, MO, USA). GW-9662 (irreversible selective PPARγ receptors antagonist) was purchased from Tocris Co. (Bristol, UK). Rat TNF-α and IL-10 ELISA kits were provided by BioSource International, Inc. (Camarillo, CA), and COX-2 ELISA kit was purchased from Abcam Biochemical Co. (Cambridge, UK). CLX and PGL were supplied by Iran Daru Pharmaceutical Co. (Tehran, Iran). Prostaglandin E2 and PPARγ ELISA kits were obtained from Crystal Day Biotech Co. (Shanghai, China). 

λ-Carrageenan was prepared by normal saline. Normal saline containing 10% DMSO was used to make the solution of CLX, GW-9662, and PGL. The route of drug administration to rats was intraperitoneal (IP) with a volume of 10 ml/kg body weight, however, carrageenan and GW-9662 were injected intra-plantarly (0.1 ml/paw, IPL). Control animals were given merely the vehicle.

***Carrageenan-induced inflammation***

The acute paw edema and inflammation were created by administration of 100 μl (IPL) carrageenan 1% solution into the right hind paw (17). Immediately before and after carrageenan injection (at 0.5, 1, 2, 3, 4, and 5 hr), the inflammation levels were measured by a plethysmometer (Ugo Basile Co., Italy). The inflammation percentage was calculated as follows (18):

Following 5 hr, rats were euthanized and the inflamed paws were cut and stored at -80 ^○^C. After taking a blood sample, the remaining serum from clotted blood was kept at -80 ^○^C for cytokine assay. Brain tissues including the cerebral cortex and hippocampus were also extracted after trans-cardiac perfusion of the rats with cold phosphate buffer saline solution and stored at -80 °C. 

***Experimental design***

In order to investigate both the maximum anti-inflammatory effects and non-effective doses of the treatments, dose-response studies were performed. CLX doses (0.3, 1, 10, and 30 mg/kg, IP), and PGL doses (1, 3, 10, and 20 mg/kg, IP) were administered 30 min before carrageenan delivery. In order to assess the association between anti-inflammatory impacts of CLX and PGL, a non-effective dose of CLX (0.3 mg/kg) and increasing doses of PGL (1, 3, and 10 mg/kg) were applied 30 min prior to carrageenan administration. Additionally, a non-effective dose of PGL (3 mg/kg) and increasing doses of CLX (0.3, 1, 10, and 30 mg/kg) were applied the same as before. Doses and time of CLX and PGL administration have been obtained from both previous experiments, particularly our previous study (19). 

To elucidate the possible role of PPARγ receptors in the observed anti-inflammatory response, CLX (10 mg/kg; IP), PGL (10 mg/kg; IP), and a combination of their non-effective doses (0.3 mg/kg CLX, 3 mg/kg PGL), pre-treated with GW-9662 (3 μg/paw IPL), were applied 15 min before drug injection. Then, after 45 min, paw edema was assessed (19-21).

***Paw homogenate preparation***

Five hours after carrageenan injection, the animals were euthanized through surgical dislocation, and the inflamed paws were removed. Tissues of rat hind-paw’s pad were collected and 5-mm pieces were homogenized in phosphate buffer solution on ice, then sonicated with an ultrasonic cell disrupter. Afterward, the prepared homogenate was centrifuged (12,000 g, 15 min, 4 °C), then the resulting supernatant was segregated and used for biochemical experiments. We used the Bradford dye-binding assay for measuring total protein amounts of rat paw tissues (22).

***PPARγ, COX-2 and PGE2 measurement***

The contents of PPARγ, COX-2, and PGE2 in paw homogenate were evaluated by ELISA kits using the manufacturer’s guidelines (23, 24). 

***Cytokine measurement***

The contents of TNF-α and IL-10 in serum were measured using ELISA kits according to the manufacturer’s guidelines (25).

***Lipid peroxidation assays***

TThe levels of lipid peroxidation, MDA contents in the cerebral cortex, hippocampus, and inflamed rat paw tissues were determined using the MDA evaluation kit (Teb Pazhouhan Razi, Tehran, Iran). In brief, the tissue homogenates were extracted and centrifuged (13000g*,* 10 min). After adding butylated hydroxytoluene to the supernatants, 100 μM mixture was added to the tubes of sodium dodecyl sulfate solution. Then, thiobarbituric acid (0.5%, w/v), sodium hydroxide, and acetic acid were added to tubes, followed by 60 min heat incubation in a boiling water bath. After cooling, the absorbance was determined at 532 nm using Synergy HT Microplate Reader (BioTek Instruments, Inc., Winooski, VT, USA). In order to determine each sample’s MDA level, a standard curve with nmol/mg protein unit was used.

***Anti-oxidant assays***

*Glutathione reduced (GSH)*

To assess the GSH contents in rats’ cortex, hippocampus, and inflamed paw, tissue homogenates were mixed with Tris–EDTA buffer (pH: 8.6) and DTNB reagent (10 mM in methanol), followed by incubation (25 °C, 20 min) which yielded a yellow color. The absorbance was read at 412 nm using a spectrophotometer (UV-1650 PC, Shimadzu, Japan). The GSH concentration of each sample was estimated by the GSH standard curve (nmol/mg protein).

*Glutathione peroxidase (GPx)*

The activity of GPx enzymes in the cortex, hippocampus, and inflamed paw tissues was determined using an enzymatic immunoassay GPx kit (Zellbio, Germany).

***Statistical analysis***

All data were presented as means±SEM Curves were graphed by plotting the Δ paw volume as a function of time. The area under the number (AUC) of Δ paw volume against time was calculated by the trapezoidal rule. Differences between treated groups were evaluated by one-way ANOVA with Tukey’s or two-way ANOVA with Bonferroni’s post-hoc tests. Statistical analysis was done by GraphPad software (GraphPad Prism 7, San Diego, CA, USA), and a *P*-value<0.05 was considered significant.

## Results

***Anti-inflammatory effects of CLX and PGL alone or in combination***

**Figure 1:**
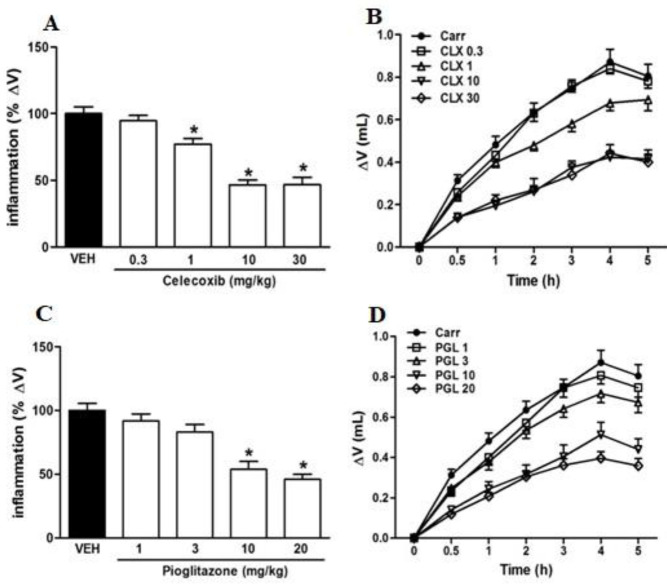
Anti-inflammatory effects of celecoxib (CLX panels A and B) and pioglitazone (PGL panels C and D) in carrageenan (Carr)-induced acute paw edema in rats. Drugs and vehicles (VEH) injection (IP) were applied 30 min before carrageenan delivery. * indicates versus vehicle-treated group (*P*<0.05). Data are expressed as Mean±SEM (one-way ANOVA followed by Tukey's test, n=7)

Carrageenan injection (IPL) increased the rat paw volume in a time-dependent manner, with the maximal volume after 4 hr (Figure 1). Both CLX and PGL significantly reduced edema in the rat paw meaning a statistically significant anti-inflammatory effect (Figures 1A-D). CLX doses (1, 10, and 30 mg/kg) showed a statistically significant anti-inflammatory effect, while the lowest dose (0.3 mg/kg) had no significant response (Figures 1A and 1B). Likewise, PGL low doses (1 and 3 mg/kg) did not result in a statistically significant anti-inflammatory effect, while at higher doses (10 and 20 mg/kg) significant anti-inflammatory responses were detected (Figures 1C and 1D). 

**Figure 2:**
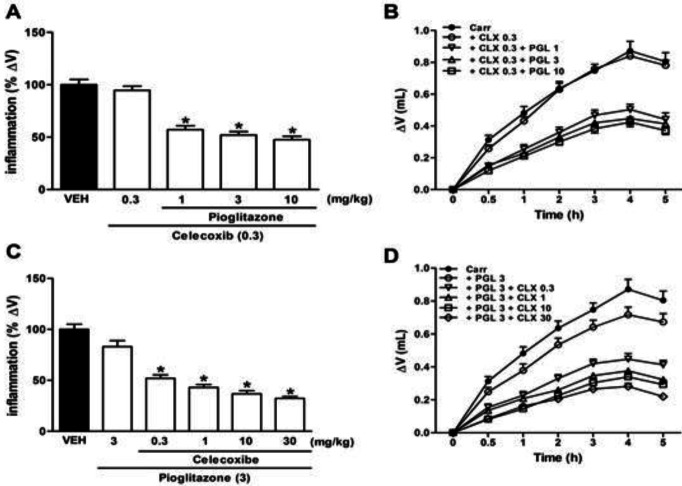
Anti-inflammatory effects of pioglitazone and celecoxib co-administration (IP) in carrageenan-induced acute paw edema in rats (panels A and B). Drugs and vehicle (VEH) injection (IP) were applied 30 min before carrageenan delivery. * indicates versus vehicle-treated group (*P*<0.05). Data are expressed as Mean±SEM (one-way ANOVA followed by Tukey's test, n=7)

In a dose-response experiment of CLX and PGL, the non-effective dose of CLX (0.3 mg/kg) was combined with increasing doses of PGL (1–10 mg/kg) and the non-effective dose of PGL (3 mg/kg) was combined with the increasing dose of CLX (0.3, 1, 10, and 30 mg/kg). These combined treatments resulted in a statistically significant anti-inflammatory response in the rat paw inflammation model (Figures 2A, 2B, 2C, and 2D *P*<0.05).

***Anti-inflammatory effects of CLX and PGL along with GW-9662***

**Figure 3:**
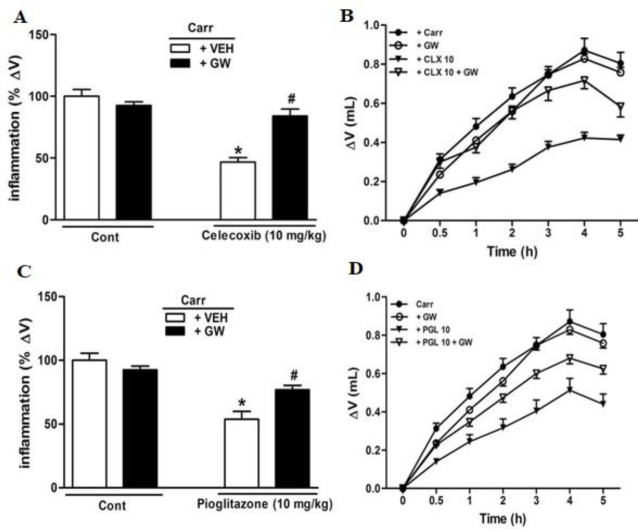
Involvement of PPARγ (Proliferator-activated receptor-gamma) receptors in the anti-inflammatory response produced by celecoxib (CLX, 10 mg/kg; IP; panels A, B) and pioglitazone (PGL, 10 mg/kg; IP; panels C, D) in carrageenan-induced acute paw edema in rats. GW-9662 (GW, IPL) was administered 15 min before carrageenan injection. * indicates versus vehicle (VEH)-treated group (*P*<0.05). # indicates versus CLX 10 and PGL 10 groups (*P*<0.05). Data are expressed as Mean±SEM (two-way ANOVA followed by Bonferroni's test, n=7)

**Figure 4:**
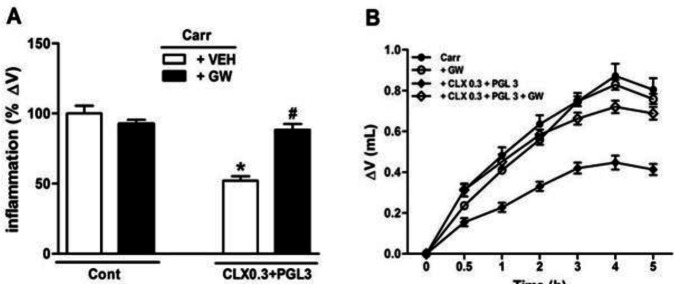
Involvement of PPARγ (Proliferator-activated receptor-gamma) receptors in the anti-inflammatory response produced by sub-effective doses of celecoxib (CLX, 0.3) and pioglitazone (PGL, 3 mg/kg) in carrageenan-induced acute paw edema in rats. GW-9662 (GW, IPL) was administered 15 min before carrageenan injection. * indicates versus vehicle (VEH)-treated group (*P*<0.05). # indicates versus CLX 0.3+PGL3 (*P*<0.05). Data are expressed as Mean±SEM (two-way ANOVA followed by Bonferroni's test, n=7)

The anti-inflammatory effects of both CLX (10 mg/kg) and PGL (10 mg/kg) were totally antagonized by GW-9662 application (3 µg/paw) into the rat inflamed paw (Figures 3 A-D). In addition, pre-treatment of GW-9662 (IPL) entirely reversed the synergistic anti-inflammatory effects of CLX+PGL doses, 5 hr following carrageenan administration (Figure 4). GW-9662 (IPL) treatment resulted in neither inflammation nor anti-inflammatory effects. 

***COX-2 alterations induced by CLX and PGL along with GW-9662***

**Figure 5:**
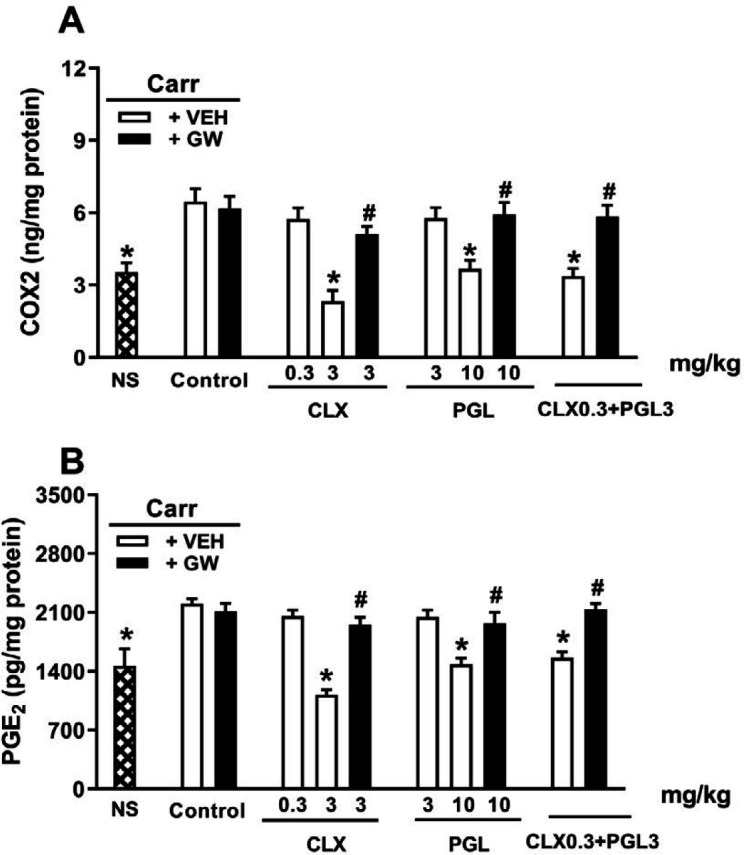
Role of PPARγ (Proliferator-activated receptor-gamma) receptors in the cyclooxygenase 2 (COX-2) (panel A) and PGE2 (panel B) alterations induced by celecoxib (CLX), pioglitazone (PGL), and their combination in carrageenan-induced acute paw edema in rats. GW-9662 (GW, IPL) was administered 15 min before carrageenan injection. * indicates versus vehicle (VE) treated group (*P*<0.05). # indicates versus its corresponding vehicle (VEH) treated group (*P*<0.05). Data are expressed as Mean±SEM (two-way ANOVA followed by Bonferroni's test, n=7)

In order to investigate the interaction between PPARγ and COX-2 levels, the impact of GW-9662 on the COX-II alterations caused by CLX and PGL alone or in combination was explored in rats. Carrageenan administration (IPL) induced a significant increase in COX-2 levels in rats (*P*<0.05). Moreover, treatment with CLX (3 mg/kg, IP), PGL (10 mg/kg, IP), and their non-effective doses together (CLX 0.3 mg/kg, PGL 3 mg/kg) caused a significant COX-2 reduction in the rat inflamed paw tissue (Figure 5A). Moreover, GW9662 pre-treatment (IPL) significantly reversed the alterations. Injection of GW-9662 alone caused no changes in COX-2 levels.

***PGE2 alterations induced by CLX and PGL along with GW-9662***

In order to investigate the interaction between PPARγ antagonism and PGE2 levels (as COX activity index), effects of GW-9662 on the PGE2 alterations caused by CLX and PGL, alone or in combination were studied. Carrageenan injection (IPL) caused a significant elevation in PGE2 levels in the inflamed rat paw (*P* < 0.05). Furthermore, treatment with CLX (3 mg/kg, IP), PGL (10 mg/kg, IP), and a combination of their non-effective doses (CLX 0.3 mg/kg, PGL 3 mg/kg) caused a significant reduction in COX-II in the rat inflamed paw (Figure 5B). However, GW9662 pre-treatment (IPL) significantly reversed the alterations in the inflamed paw. Injection of GW-9662 alone caused no changes in the PGE-2 levels. 

***PPARγ alterations induced by CLX and PGL along with GW-9662***

**Figure 6:**
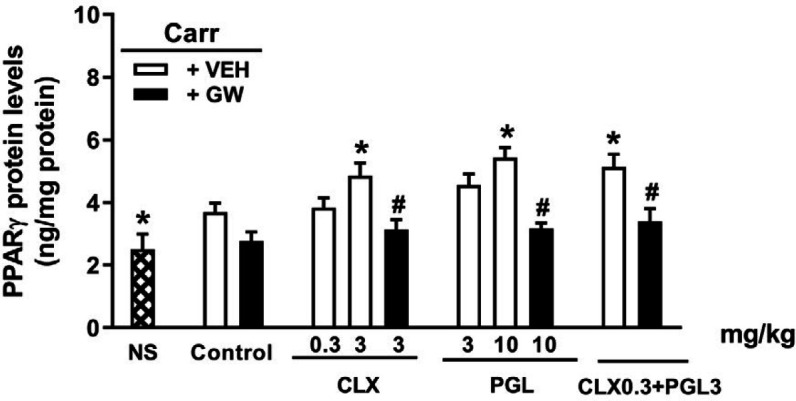
Effect of PPARγ (Proliferator-activated receptor-gamma) receptor inhibition on tissue PPARγ alterations produced by celecoxib (CLX), pioglitazone (PGL), and their combination in carrageenan-induced acute paw edema in rats. GW-9662 (GW, IPL) was administered 15 min before carrageenan injection. * indicates versus vehicle (VE) treated group (*P*<0.05). # indicates versus its corresponding vehicle (VEH) treated group (*P*<0.05). Data are expressed as Mean±SEM (two-way ANOVA followed by Bonferroni's test, n=7)

In order to investigate the interaction of COX-2 inhibition with PPARγ, effect of GW-9662 on PPARγ alterations caused by CLX and PGL, alone or in combination was evaluated in the rat inflamed paws. Carrageenan administration (IPL) resulted in a significant rise in PPARγ amounts (*P*<0.05). Moreover, treatment with CLX (3 mg/kg, IP), PGL (10 mg/kg, IP), and combination of their non-effective doses (CLX 0.3 mg/kg, PGL 3 mg/kg) caused a significant elevation in the PPARγ levels in rats (Figure 6). However, GW9662 pre-treatment significantly reversed the alterations in PPARγ. Injection of GW-9662 alone caused no change to PPARγ levels.

***Cytokine alterations induced by CLX and PGL along with GW-9662***

**Figure 7:**
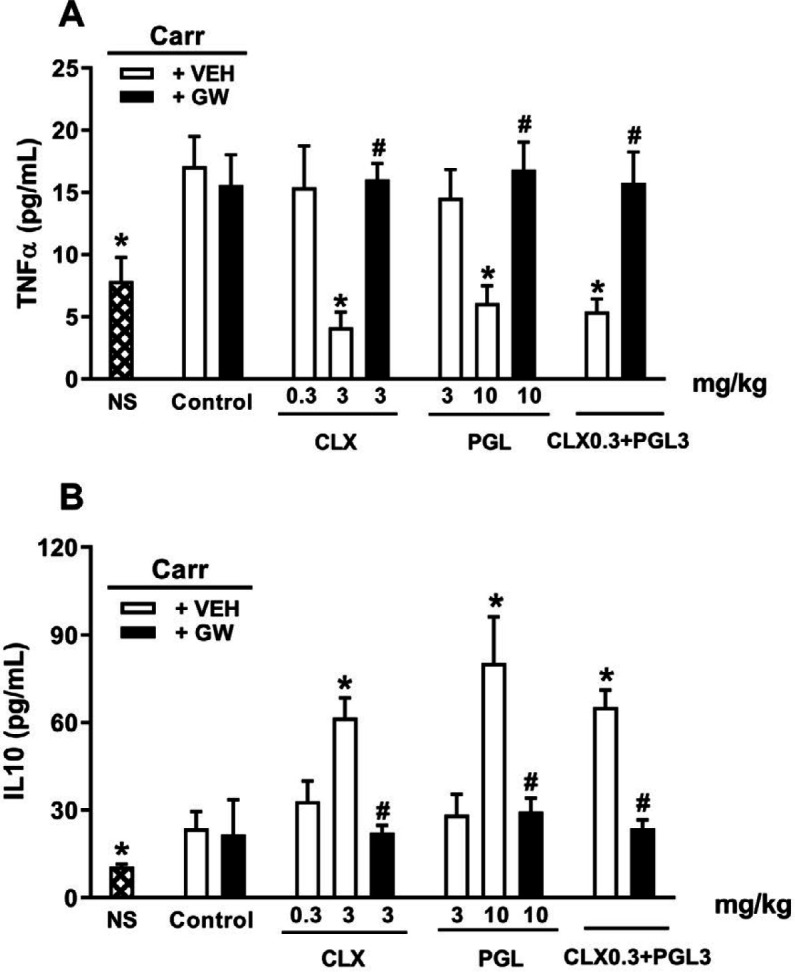
Role of PPARγ receptors in serum levels of TNF-α (panel A) and IL-10 (Panel B) alterations induced by celecoxib (CLX), pioglitazone (PGL), and their combination in carrageenan-induced acute paw edema in rats. GW-9662 (GW, IPL) was administered 15 min before carrageenan injection. * indicates versus vehicle (VE) treated group (*P*<0.05). # indicates versus its corresponding vehicle (VEH) treated group (*P*<0.05). Data are expressed as Mean±SEM (two-way ANOVA followed by Bonferroni's test, n=7)

Carrageenan administration (IPL) caused a significant elevation in serum contents of TNF-α and IL-10 (*P*<0.05). In addition, treatment with CLX (3 mg/kg, IP), PGL (10 mg/kg, IP), and a combination of their non-effective doses (CLX 0.3 mg/kg, PGL 3 mg/kg) caused a significant reduction in serum amounts of TNF-α, and a noticeable rise in serum IL-10 (*P*<0.05, Figure 7). However, GW9662 pre-treatment significantly reversed the alterations in both serum TNF-α and IL-10. Injection of GW-9662 exclusively showed no effects on these two cytokines. 

***MDA, GSH, and GPx alterations induced by CLX and PGL along with GW-9662***

Treatment with anti-inflammatory effective doses of CLX (3 mg/kg) and PGL (10 mg/kg) noticeably decreased the levels of MDA, while increasing GSH levels and GPx activity in the rat brain cortex, hippocampus, and inflamed paw tissues (*P*<0.05). Interestingly, all observed effects were antagonized by GW-9662 injection (IPL) (*P*<0.05; Table 1).

**Table 1:** Role of PPARγ receptors in malondialdehyde (MDA), reduced glutathione (GSH) and glutathione peroxidase (GPx) alterations induced by celecoxib (CLX), pioglitazone (PGL), and their combination in carrageenan-induced acute paw edema in rats. GW-9662 (GW, IPL) was administered 15 min before carrageenan injection

	**Paw**			**Cortex**			**Hippocampus**		
	**MDA **	**GSH**	**GPx**	**MDA **	**GSH**	**GPx**	**MDA**	**GSH**	**GPx**
**VEH**	**3.4±0.45**	**30.4±1.35**	**7.1±0.85**	**3.9±0.48**	**22.5±1.75**	**6.7±0.61**	**2.8±0.26**	**25.3±1.94**	**7.8±0.58**
**CLX3 + VEH **	**1.4±0.30** ^*^	**43.7±2.34** ^*^	**10.7±1.19** ^*^	**2.8±0.28** ^*^	**28.1±2.04** ^*^	**8.2±0.74** ^*^	**2.1±0.20** ^*^	**31.1±2.65** ^*^	**9.8±0.75** ^*^
**CLX3 + GW**	**3.1±0.59** ^#^	**36.3±2.00** ^#^	**8.7±0.95** ^#^	**3.4±0.51**	**26.4±1.72**	**7.5±0.68**	**2.6±0.29** ^#^	**28.3±1.82**	**8.6±0.54**
**PGL10 + VEH**	**1.4±0.20** ^*^	**41.5±3.25** ^*^	**11.2±1.62** ^*^	**2.6±0.20** ^*^	**31.4±2.13** ^*^	**7.8±0.52** ^*^	**2.4±0.26**	**29.1±2.95** ^*^	**10.1±0.92** ^*^
**PGL10 + GW**	**3.5±0.64** ^#^	**35.4±2.64** ^#^	**8.2±1.24** ^#^	**3.6±0.44** ^#^	**25.3±2.04** ^#^	**7.2±0.74**	**2.8±0.23**	**28.2±2.09**	**9.2±0.85**
**GW**	**3.1±0.35**	**32.5±1.21**	**7.4±0.61**	**3.8±0.38**	**21.9±1.43**	**6.9±0.54**	**2.7±0.25**	**24.4±1.68**	**8.1±0.67**

* indicates versus vehicle (VEH)-treated group (*P*<0.05). # indicates versus CLX 3, and PGL10 treated groups (*P*<0.05). Data are expressed as Mean±SEM (two-way ANOVA followed by Bonferroni's test, n=7)

MDA: nmol/mg protein; GSH: nmol/mg protein, GPx: U/mg protein

## Discussion

The current study proved the anti-inflammatory effects of CLX induced by down-regulating the inflammatory cytokines and oxidative stress through PPARγ receptors in the carrageenan model of inflammation in rats. 

Inflammation is the immune system’s response to tissue injury and has been implicated in the pathogeneses of many conditions such as neurodegenerative and cardiovascular diseases (26). Paw edema induced by carrageenan is a valid model of inflammation with several inflammatory biochemicals as its development factors. The present study showed that CLX produced dose-dependent anti-inflammatory effects against carrageenan-induced paw edema in rats. Our results confirmed the previous findings concluding that CLX exerted noticeable effects in inflammatory conditions (27).

COX-II isoenzyme has a crucial role in inflammatory processes. Increased expression of COX-II isoenzyme has been detected in many inflammatory events leading to biosynthesis of prostaglandins. In addition, the role of PPARγ has been reported in COX-II metabolic signaling pathways. On the other hand, there is a cross-talk between these two important mediators in such a way that they could synergistically suppress some diseases like cancer (28). In the current study, we showed that a non-effective dose of CLX was potentiated by PGL and vice versa in acute inflammation induced by carrageenan, indicating the synergistic interaction between COX-II and PPARγ pathways. It is worth noting that these effects were antagonized by an irreversible inhibitor of PPARγ receptors, confirming the interaction. In agreement with the mentioned effects, we observed that CLX suppressed COX-II and prostaglandin E_2_ in the rat inflamed paw, which were antagonized by GW-9662. Furthermore, administration of CLX increased the level of PPARγ receptors in the inflamed paw tissues. With this regard, we previously reported that IND could be able to cause the same results in the rat model of acute inflammation (19).

Carrageenan administration to the subplantar surface of the rat paw elevates the release of TNF-α as one of the important pro-inflammatory cytokines. TNF-α triggers the release of IL-1β and cytokine-induced neutrophil chemo-attractant 1. These mediators are responsible for the stimulation of the PGs synthesis by COX-II (29). Additionally, the activation of PPARγ receptors could down-regulate cytokine-stimulated COX-II expression via inhibition of NF-κB (nuclear factor kappa-light-chain-enhancer of activated B cells) or other transcription mediators (30). Results of the current study demonstrated that CLX suppressed inflammatory cytokines, which were potentiated by PGL and reversed by GW-9662, suggesting the role of PPARγ receptors. Furthermore, IL-10 is an anti-inflammatory cytokine that suppresses COX-II expression and consequently PGE_2_ production (31). Kim *et al.* (2005) reported the role of PPARγ on immune-regulatory effects of IL-10 (32). Thus, our results indicated that CLX increased serum levels of IL-10 through PPARγ receptors.

Oxidative stress is caused as a result of the disparity between the reactive oxygen species (ROS) output during metabolism and their elimination by antioxidant defense systems. It is known that oxidative stress has a close connection with inflammatory processes (33). In this regard, it has been shown that ROS has a role in the generation of cytokines through modulating NF-κB, which could regulate their synthesis (34). Moreover, ROS promotes the recruitment of endothelial cells into the inflammation site through the induction of adhesion molecules, and finally produces more tissue damages by direct interactions with different biomolecules (35). Evidence has suggested potential associations between oxidative stress and induction of COX-II resulting in prostaglandin generation (36). Furthermore, free radicals have been demonstrated to have an essential role in carrageenan-induced acute inflammation (37). In the current work, we proved that CLX reduced oxidative stress by improving endogenous antioxidant mechanisms in peripheral and central tissues through PPARγ receptors in carrageenan-induced inflammation. In this regard, Amic *et al.* (2016) reported that polyphenolic compounds with COX-2 inhibitory effects might contribute to the suppression of oxidative stress (38). Additionally, Collino *et al.* (2006) reported the modulatory role of PPARγ receptors in oxidative stress and inflammatory responses in forebrain ischemia-reperfusion injury in rats (39).

## Conclusion

In summary, we found that CLX along with PGL prevented acute local inflammation caused by carrageenan in rats. This effect of CLX seems to have resulted from inhibition of inflammatory cytokines and oxidative stress. Moreover, we found that PPARγ receptors could be contributed to the anti-inflammatory effects of CLX. The findings of the current study suggest that a combination of CLX and PGL might be used in the management of acute inflammation particularly in diabetic populations. 
